# Effectiveness of Systemic Insecticide Dog Treatment for the Control of Chagas Disease in the Tropics

**DOI:** 10.3390/biology12091235

**Published:** 2023-09-13

**Authors:** Edem Fiatsonu, Aniruddha Deka, Martial L. Ndeffo-Mbah

**Affiliations:** 1 Department of Veterinary Integrative Biosciences, College of Veterinary Medicine and Biomedical Sciences, Texas A&M University, College Station, TX 77845, USA; aniruddhad9@exchange.tamu.edu (A.D.); m.ndeffo@tamu.edu (M.L.N.-M.); 2 Department of Epidemiology and Biostatistics, School of Public Health, Texas A&M University, College Station, TX 77845, USA

**Keywords:** *Trypanosoma cruzi*, chagas disease, systemic insecticide, Ross–MacDonald model

## Abstract

**Simple Summary:**

Chagas disease, a vector-borne disease caused by the parasite *Trypanosoma cruzi*, is a significant threat to human and canine health in the tropics. To control the transmission of *T. cruzi*, systemic insecticide treatment of dogs with fluralaner has been proposed as an intervention for canine and potentially human Chagas disease. In this study, we evaluated the efficacy of canine treatment regimens with fluralaner to reduce Chagas disease infections (once every three months and once every twelve months) in high and low endemic regions using a data-driven mathematical model. Our study shows that Fluralaner treatment can effectively reduce *T. cruzi* transmission in humans, but may increase infections in dogs if canine consumption of triatomine increases. The effectiveness of the treatment regimen was shown to vary substantially with the underlying intensity of *T. cruzi* transmission and the increased rate of canine consumption of dead triatomines. Our study provides new evidence to support further empirical studies on the potential impact of mass treatment of dogs with systemic insecticides as a novel and additional intervention for the control and elimination of Chagas disease in the tropics.

**Abstract:**

Chagas disease, caused by *Trypanosoma cruzi* and transmitted by triatomines, can lead to severe cardiac issues and mortality in many mammals. Recent studies have shown that systemic insecticide treatment of dogs is highly effective in killing triatomines. Here, we assessed the impact of dog treatment on *T. cruzi* transmission. We developed a mathematical model of *T. cruzi* transmission among triatomines, dogs, humans, and rodents. We used the model to evaluate the impact of dog treatment regimens on *T. cruzi* transmission dynamics to determine their effectiveness in reducing *T. cruzi* infection among hosts. We show that a 3-month treatment regimen may reduce *T. cruzi* incidence among humans by 59–80% in a high transmission setting, and 26–82% in a low transmission setting. An annual treatment may reduce incidence among humans by 49–74% in a high transmission setting, and by 11–76% in a low transmission setting. However, dog treatment may substantially increase *T. cruzi* prevalence among dogs if dog consumption of dead triatomines increases. Our model indicates that dog treatment may reduce *T. cruzi* infections among humans, but it may increase infections in dogs. Therefore, a holistic approach targeting different hosts is necessary for Chagas elimination.

## 1. Introduction

Chagas disease is a vector-borne neglected tropical disease that poses a significant health burden in tropical regions [1]. The parasite is primarily transmitted by triatomine insects, commonly known as “kissing bugs”, via contact with their infected fecal material during or after feeding. In addition, transmission can occur when the host ingests the infected bugs or their feces [2,3]. Vertical transmission through transplacental transmission or breast milk, and other routes such as direct contact with infected body fluids, blood transfusion, and organ transplantation, also contribute to parasite transmission [4]. The causative agent of Chagas disease is the parasite *Trypanosoma cruzi*, and unfortunately, specific treatment options are limited, and viable vaccines are still lacking. Originally endemic in the Americas, spanning from Chile to the United States of America, the disease has now become a global health concern due to human migration, resulting in a significant number of cases in non-endemic regions such as Canada and Europe [5,6]. There are approximately 6 million reported human cases in the Americas alone, but the true disease burden may be even higher due to differences in disease surveillance and reporting practices between countries [1]. For example, recent estimates for Mexico, one of the most affected countries, range from less than 1 million to more than 4 million cases, and there is considerable uncertainty in these figures, possibly influenced by reporting bias [7,8,9]. Chagas disease particularly affects vulnerable communities and can lead to severe cardiac problems. The global annual public health impact of the disease is approximately $627.46 million in healthcare costs and 806,170 disability-adjusted life years (DALYs) lost due to both mortality and morbidity [10]. These statistics underscore the urgent need for effective and comprehensive strategies to control and manage Chagas disease on a global scale.

Triatomines are blood-sucking insects of the family Reduviidae found primarily in the Americas. There are more than 150 species of triatomines distributed across different tribes, with diverse morphological characteristics and unique wing patterns [11,12]. Species such as *Rhodnius prolixus*, *Triatoma infestans*, and *Triatoma dimidiata* are of significant medical importance due to their high prevalence, wide distribution, and efficient transmission of Chagas disease. Understanding the biology, distribution, and vectorial capacity of these triatomine species is critical for effective disease control and prevention strategies [11]. In tropical regions such as Latin America, triatomine species such as *T. infestans*, *T. dimidiata*, and *R. prolixus* are widespread and well-adapted to human dwellings, contributing to Chagas disease transmission [12]. These species exhibit a high degree of domiciliation in and around human habitats [13,14,15]. These triatomine species are commonly found in rural areas with suitable housing conditions and animal reservoirs [14,15].

Efforts to control and eradicate Chagas disease have concentrated on targeting triatomine bugs, the insect vectors [12]. Specifically, *T. cruzi* transmission to humans occurs via two primary cycles: the domestic and peri-domestic cycles [16]. In the peri-domestic cycle, small wild mammals serve as reservoirs, with peri-domestic bugs introducing the parasite into households and infecting humans and domesticated mammals. The domestic cycle involves the colonization of houses by triatomine bugs, which in certain regions facilitates transmission between humans and domesticated mammals [16]. The epidemiological importance of rodents becomes even more apparent when considering that many species can also enter human dwellings and contribute to the transmission of *T. cruzi* infections [17]. Given the presence of numerous wild mammalian reservoirs, elimination of the disease in the peri-domestic cycle is very challenging [18,19]. However, within the domestic cycle, dogs serve as more accessible reservoirs than wild animals, providing opportunities for One Health interventions to target *T. cruzi* transmission and reduce human Chagas disease [20]. Dogs have been identified as a major contributor to *T. cruzi* transmission [21]. These animals play an increasingly important role in societies, serving as pets, companions, guard dogs, hunting partners, herding assistants, and law enforcement aids [22]. Dogs can contribute to the transmission of *T. cruzi* in several ways. They may ingest infected triatomine, thereby maintaining the transmission cycle of the parasite as a primary or major reservoir host. In addition, dogs can contribute to the proliferation of triatomine bug populations by serving as an important source of blood meals [23]. Consequently, dogs can establish a link between the peridomestic and domestic transmission cycles, increasing the risk of human *T. cruzi* infection [24,25,26].

In the tropics, particularly in Argentina, several studies have demonstrated and documented the significant involvement of dogs in areas endemic to *T. cruzi* [24,27,28,29]. In the northwestern region, dogs exhibited a notably higher infection rate (65.1%) compared to that of humans (34.2%). In addition, dogs were found to be 18 times more infectious to *T. infestans* than humans [27]. Furthermore, *T. infestans*, a major vector of *T. cruzi* in the tropics, consistently shows a preference for dogs over other domestic animals [22]. This strong preference for triatomine vectors for dogs can be exploited via a targeted vector control approach known as *xenointoxication*. In this strategy, pesticides are applied to peri-domestic and domestic animals, such as dogs, with the goal of suppressing insect infestations. By specifically targeting dogs with topical insecticides or insecticide-impregnated collars, dogs effectively become baited lethal traps [20,30]. In the context of a pyrethroid shortage, the administration of a safe, long-lasting, and effective insecticide such as fluralaner to dogs could potentially serve as a valuable resource to interrupt the transmission of *T. cruzi* [20]. Interventions targeting the dog population to interrupt *T. cruzi* transmission in the domestic cycle have been evaluated. Mathematical models suggest that removal of infected dogs from households with infected humans could interrupt disease transmission but culling the dog population is socially unacceptable [31]. Recent trials of oral or topical insecticides, particularly fluralaner, have shown promising efficacy in killing triatomines that feed on dogs [30,32,33,34]. These interventions have the potential to be cost-effective in reducing *T. cruzi* infection in humans [35]. Annual treatment of dogs with fluralaner may be effective in reducing infection rates in high transmission settings, but caution is needed in low transmission settings [36]. However, there is a potential counterproductive effect as dogs may ingest treated infected triatomines, increasing infection rates in the dog population.

The Ross-MacDonald theory has made significant contributions to the advancement of quantitative theory and basic principles of epidemiology, particularly in the context of vector-borne diseases [37,38]. Mathematical models have been developed to study and better understand the dynamics of *T. cruzi* transmission with an emphasis on protecting human health [36,39,40,41,42,43]. Mathematical modeling techniques provide valuable insights into the cycle of *T. cruzi* transmission and the potential impacts of host-targeted interventions. In this study, we modified the traditional Ross-MacDonald model to examine the dynamics of transmission among triatomines, dogs, rodents, and humans in domestic and peri-domestic settings. We used the model to evaluate the effectiveness of treating dogs with different treatment strategies using the systemic insecticide fluralaner to control triatomine populations and reduce *T. cruzi* infections among hosts. In addition, we considered the potential impact of increased triatomine consumption and the risk of oral transmission when fluralaner is administered regularly to dogs.

## 2. Materials and Methods

We conducted a simulation study by modifying the traditional Ross-MacDonald model. Our adapted model made several simplifying assumptions. First, we assumed that the population of hosts (humans, dogs, and other competent hosts, i.e., rodents) was homogeneous and remained constant throughout the study. Similarly, we assumed that the vector population (triatomine) was also homogeneous but differed from the classic Ross-MacDonald model by incorporating a logistic birth rate for triatomine [44]. We based the parameters of the triatomine population on data related to *T. infestans*, the primary vector of *T. cruzi* transmission in the tropics. In addition, we assumed that both triatomine bugs and hosts (humans, dogs, and other competent hosts) can be susceptible (*S*, not infected with *T. cruzi* and able to become infected) or infectious (*I*, infected with *T. cruzi* and able to transmit). We denoted by $N_{H},\;N_{D},\;N_{O}$ and $N_{V}$, the population sizes of humans, dogs, other competent (rodents), and triatomines, respectively. We denoted $S_{V}$ and $\underline{I_{V}}$ as the number of susceptible and infected triatomines and $\beta_{VH},\beta_{VD},$ and $\beta_{VO}$ are the transmission rate of *T. cruzi* from humans, dogs, and other competent hosts to triatomine. The number of triatomine births is determined by the birth rate R, carrying capacity κ and the total number of triatomines$\;N_{V}$ in each setting using the following formula:

$$R\left(N_{V}\right)\left(1-\frac{N_{V}}{\kappa }\right).$$

**Figure 1:**
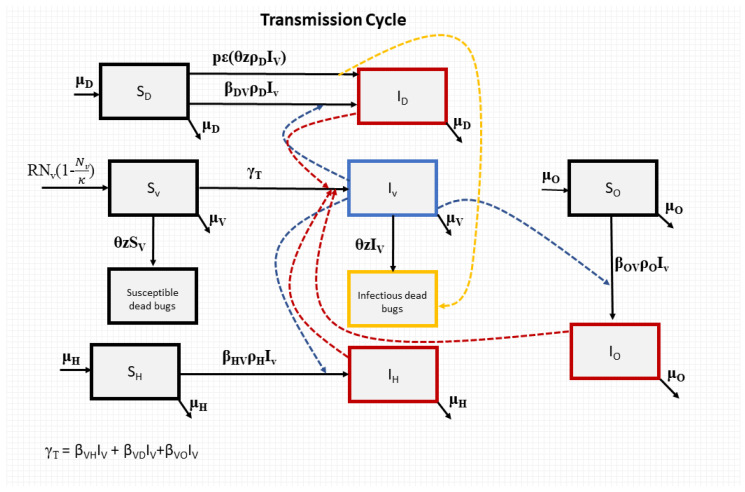
Flowchart for our model of *T. cruzi* transmission. The blue, red, and yellow dotted lines represent the transmission processes of *T. cruzi*.

The model flow diagram is provided in Figure 1.

### 2.1. Human, Dogs, and Other Transmitters (Rodents)

We denoted $\beta_{HV},\beta_{DV}$ and $\beta_{OV}$ as the transmission rate of *T. cruzi* from triatomine to humans, dogs, and other competent hosts, respectively. The transition of hosts (humans, dogs, and rodents) from a susceptible state to an infectious state occurs at a rate determined by the force of infection (FOI) resulting from vector-borne transmission. This FOI is calculated as the product of various factors, including the transmission rate of *T. cruzi* from triatomine to hosts, the ratio of triatomine associated with humans, dogs, and other competent hosts as $\rho_{H}$, $\rho_{D\;}$and $\rho_{O}$, respectively, and the proportion of infected triatomines $\underline{I_{V}}$ present in the system. We further denote by infected hosts by *I_H_*, *I_D_*, and *I_O_* the proportion of humans, dogs, and other competent hosts (rodents) infected with *T. cruzi* and able to transmit. Prior to dog treatment, disease transmission dynamics between the hosts (humans, dogs, and other competent hosts (rodents)) and the triatomine are described and presented by the following system of nonlinear ordinary differential equations: $dI_{H}$, the change in the proportion of infected humans, $dI_{D}$, the change in the proportion of infected dogs, $dI_{O}$, the change in the proportion of infected other competent hosts (rodents), $d\underline{S_{V}}$, the change in the proportion of susceptible triatomines and $d\underline{I_{V}}$, the change in the proportion of infectious triatomines:

$$dI_{H}=\beta_{HV}\rho_{H}\left(1-I_{H}\right)\left(\frac{\underline{I_{V}}}{N_{V}}\right)-\mu_{H}I_{H}$$

$$dI_{D}=\beta_{DV}\rho_{D}\left(1-I_{D}\right)\left(\frac{\underline{I_{V}}}{N_{V}}\right)-\mu_{D}I_{D}$$

$$dI_{O}=\beta_{OV}\rho_{O}\left(1-I_{O}\right)\left(\frac{\underline{I_{V}}}{N_{V}}\right)-\mu_{O}I_{O}$$

$$d\underline{S_{V}}=RN_{V}\left(1-\frac{N_{V}}{\kappa }\right)-\left(\beta_{VH}I_{H}+\beta_{VD}I_{D}+\beta_{VO}I_{O}\right)\underline{S_{V}}-\mu_{V}\underline{S_{V}}$$

$$d\underline{I_{V}}=\left(\beta_{VH}I_{H}+\beta_{VD}I_{D}+\beta_{VO}I_{O}\right)\underline{S_{V}}-\mu_{V}\underline{I_{V}}$$

where $N_{V}=\underline{S_{V}}+\underline{I_{V}}$.

$\rho_{H}$, $\rho_{D\;}$and $\rho_{O}$ are the ratio of triatomine to humans, dogs, and other competent hosts, and are estimated as 9.3:1; 31.6:1, and 13.7:1, respectively [2,45]. The model’s parameters and corresponding values are provided in Table 1.

In the absence of *T. cruzi* infection, triatomine carrying capacity is derived as follows:

At equilibrium, $dN_{V}$ = 0, thus

$$dN_{V}=RN_{V}\left(1-\frac{N_{V}}{\kappa }\right)-\mu_{V}N_{V}=0,\;\mathrm{and}\;\kappa =\frac{RN_{V}}{R-\mu_{V}}$$

**Figure 2:**
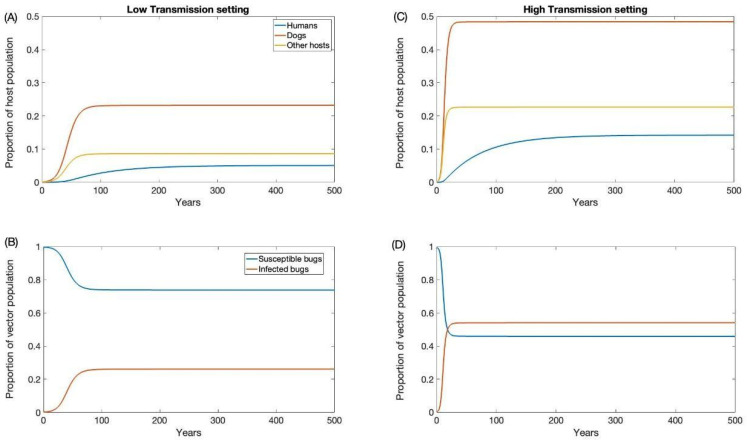
Endemic equilibrium for low (**A**,**B**) and high (**C**,**D**) transmission settings.

For our high and low prevalence settings, the endemic prevalence was estimated to be 0.54 and 0.26 for triatomine, 0.48 and 0.23 for dogs, 0.22 and 0.08 for other hosts, and 0.14 and 0.05 for humans, respectively. These estimates are consistent with empirical estimates from the tropics [54,55,56,57,58]. To initialize our simulations at endemic prevalence in each transmission setting, our model was run long enough to reach equilibrium (Figure 2).

### 2.2. Dog Treatment

Fluralaner, an oral systemic insecticide, is used in dogs to prevent tick and flea infestations. Fluralaner-treated dogs have been shown to effectively kill triatomine feeding on them [34,59]. The triatomine mortality rate induced from dog treatment was defined as θz where θ is the triatomine contact rate with dogs, with $\theta =\frac{\beta_{VD}}{b_{VD}}$, where $b_{VD}$ is the probability of triatomine infection per feed on *T. cruzi* infected dogs and z is the percentage of bugs that will die after feeding on treated dogs at that given time point. Temporal changes in z were informed using empirical data from systematic laboratory studies [34,59].

As dog treatment results in a substantial increase in dead triatomines, this may change the force of infection in dogs due to the potential ingestion of dead infected bugs. We considered this oral force of infection in our model via the additional transmission factor $p\varepsilon \left(\theta z\rho_{D}\right)\left(\frac{\underline{I_{V}}}{N_{V}}\right)$ where p is the proportion of dead triatomines consumed by dogs and ε is the probability of dog infection via triatomine ingestion. The system of equations becomes the following:

$$dI_{H}=\beta_{HV}\rho_{H}\left(\frac{\underline{I_{V}}}{N_{V}}\right)\left(1-I_{H}\right)-\mu_{H}I_{H}$$

$$dI_{D}=(\beta_{DV}\rho_{D}+p\varepsilon \left(\theta z\rho_{D}\right)\left(\frac{\underline{I_{V}}}{N_{V}}\right)\left(1-I_{D}\right)-\mu_{D}I_{D}$$

$$dI_{O}=\beta_{OV}\rho_{O}\left(\frac{\underline{I_{V}}}{N_{V}}\right)\left(1-I_{O}\right)-\mu_{O}I_{O}$$

$$d\underline{S_{V}}=RN_{V}\left(1-\frac{N_{V}}{\kappa }\right)-\left(\beta_{VH}I_{H}+\beta_{VD}I_{D}+\beta_{VO}I_{O}\right)\underline{S_{V}}-\mu_{V}\underline{S_{V}}-\theta z\underline{S_{V}}$$

$$d\underline{I_{V}}=\left(\beta_{VH}I_{H}+\beta_{VD}I_{D}+\beta_{VO}I_{O}\right)\underline{S_{V}}-\mu_{V}\underline{I_{V}}-\theta z\underline{I_{V}}$$

Using our model, we assessed the effectiveness of both regimens in reducing the prevalence of *T. cruzi* infection in dogs and triatomines, as well as the density of triatomines in two transmission settings: high and low. The effective outcomes we considered include the reduction in *T. cruzi* prevalence in various hosts (dogs, humans, and other competent hosts like rodents), the decrease in triatomine density, and the reduction in *T. cruzi* incidence in humans.

### 2.3. Treatment Strategies

In this study, we examined two distinct fluralaner treatment approaches: a 3-month regimen, where dogs received treatment once every three months and a 12-month regimen. The efficacy of each treatment and the induced triatomine mortality rate were determined based on empirical data [34]. Our models were utilized to assess the effectiveness of both regimens in reducing *T. cruzi* infection prevalence among dogs, humans, other hosts (rodents), and triatomine, and reduction in human infection incidence, in two transmission settings (high and low). To evaluate the potential impact of increased oral transmission on the effectiveness of the dogs’ treatment, we considered varying levels of dead triatomine consumption by dogs (low, medium, and high). These dog consumption levels were defined as Low, p=0.01; Medium, p=0.2; and High, p=0.6, where p is the proportion of dead triatomines consumed by dogs.

All analyses for the figures were performed in MATLAB 2022b. In our model, treatment was initiated once the population of dogs, humans, other competent hosts, and the vector population reached equilibrium.

## 3. Results

In our study, we investigated two distinct dog treatment regimens targeting the domestic vector of Chagas disease. The domestic vector refers to the triatomine insects that have adapted to living in and around human dwellings and play a significant role in transmitting the *T. cruzi* parasite responsible for Chagas disease. The two treatment regimens involved administering canine fluralaner treatment every three months and annually for a period of 20 years. The frequent treatment approaches aimed to evaluate the long-term effects of a less frequent intervention strategy on the prevalence of *T. cruzi* infection in the dog population. Additionally, we explored its potential implications for Chagas disease transmission to humans and other competent hosts.

**Figure 3:**
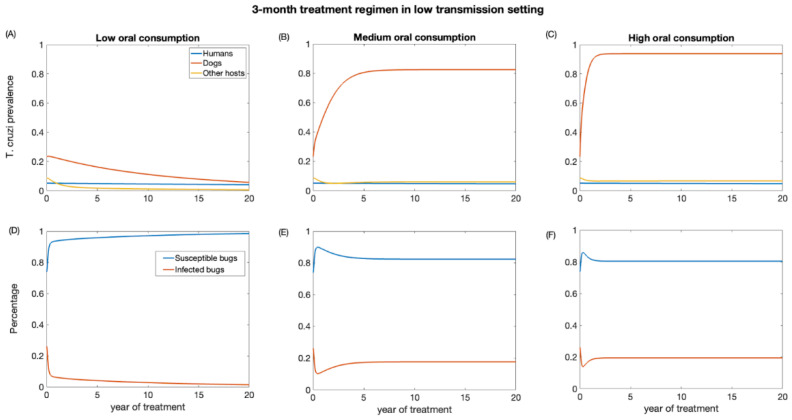
Impact of systemic insecticide treatment of dogs with fluralaner for the control of Chagas disease in low transmission setting with a 3-month regimen under different levels of dog consumption of dead triatomines. (**A**,**D**) Low oral consumption, (**B**,**E**) medium oral consumption, and (**C**,**F**) high oral consumption.

**Figure 4:**
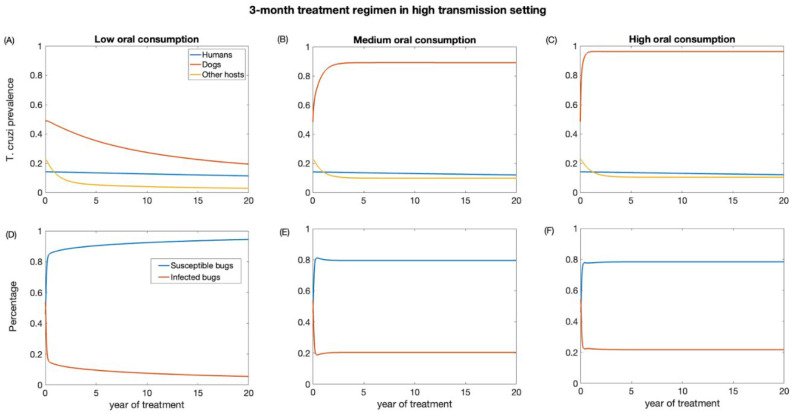
Impact of systemic insecticide treatment of dogs with fluralaner for the control of Chagas disease in high transmission setting with a 3-month regimen under different levels of dog consumption of dead triatomines. (**A**,**D**) Low oral consumption, (**B**,**E**) medium oral consumption, and (**C**,**F**) high oral consumption.

First, we examined the impact of a 3-month treatment regimen in both low- and high-transmission settings. These scenarios were evaluated for different levels (low, medium, and high) of dead triatomine consumption by dogs. As the percentage of dead triatomines eaten by dogs increases, the effectiveness of dog treatment for reducing *T. cruzi* infection decreases. The impact on reducing *T. cruzi* prevalence in the human population is marginal, primarily because humans have a longer lifespan, and our study considered a relatively short period of only 20 years (Figure 3 and Figure 4). In the low-transmission setting, the prevalence of *T. cruzi* decreased from 0.05 to 0.04, 0.046, and 0.047 in humans for low, medium, and high oral consumption of triatomines over 20 years, respectively (Figure 3), whereas in the high-transmission setting, the prevalence of *T. cruzi* decreased from 0.14 to 0.11, 0.12, and 0.12 in humans with low, medium, and high oral consumption of triatomines, respectively (Figure 4). Similarly, for other host populations, the prevalence of *T. cruzi* in the low-transmission setting decreased from 0.08 to 0.005, 0.059, and 0.065 for a low medium, and high oral consumption of triatomines, respectively (Figure 3). On the other hand, in a high transmission setting, *T. cruzi* prevalence in the other host (rodents) population decreased from 0.22 to 0.03, 0.09, and 0.1 with low, medium, and high oral consumption of triatomines, respectively (Figure 4). *T. cruzi* prevalence in both the dog and other host population shows a consistent decline for low consumption of dead triatomines in both low and high transmission settings (Figure 3 and Figure 4). In the low transmission setting, when the percentage of dead triatomines consumed is lower (1%), the level of *T. cruzi* prevalence decreases from 0.23 to 0.05, while in the high transmission setting, it decreases from 0.48 to 0.19 among the dog population.

However, when the consumption of dead triatomines is medium and higher (20% and 60%), the level of *T. cruzi* prevalence among dogs increases in both transmission settings (Figure 3 and Figure 4). With a medium oral consumption of dead triatomines by dogs, *T. cruzi* prevalence among dogs rises from 0.23 to 0.82 in a low-transmission setting and from 0.48 to 0.89 in a high-transmission setting. Similarly, for higher oral consumption of dead triatomines, *T. cruzi* prevalence increases from 0.23 to 0.93 in the low-transmission setting and from 0.48 to 0.96 in the high transmission setting.

Our analysis shows that canine fluralaner treatment administered every 3 months had an immediate impact in reducing the percentage of infected triatomine population across both transmission settings (Figure 3 and Figure 4). This impact was observed even under various levels of oral consumption of dead triatomines by dogs. In the low transmission setting, when dogs had a low level of oral consumption of triatomines, we observed a continuous decline in the percentage of infected triatomine population over the 20-year period, decreasing from 25% to 1%. Similarly, in the high transmission setting, with a low level of oral consumption of triatomines, the percentage of the triatomine population also exhibited a decreasing trend, declining from 53% to 5% over the 20-year period. On the other hand, under the medium and higher consumption of dead triatomines by dogs in both settings, we see a steep decline in the percentage of the infected triatomine population immediately after treatment, which then gradually increased and eventually stabilized. With a medium oral consumption of dead triatomines by dogs, the infected triatomine population decreases from 25% to 17% in a low-transmission setting and from 53% to 20% in a high transmission setting. Similarly, for higher oral consumption of dead triatomines, *T. cruzi* prevalence decreases from 25% to 21.6% in the low-transmission setting and from 53% to 19.5% in the high transmission setting.

**Figure 5:**
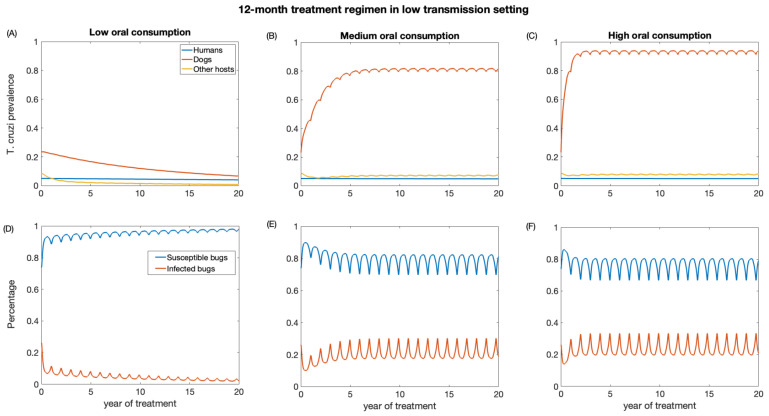
Impact of systemic insecticide treatment of dogs with fluralaner for the control of Chagas disease in low transmission setting with a 12-month regimen under different levels of dog consumption of dead triatomines. (**A**,**D**) Low oral consumption, (**B**,**E**) medium oral consumption, and (**C**,**F**) high oral consumption.

**Figure 6:**
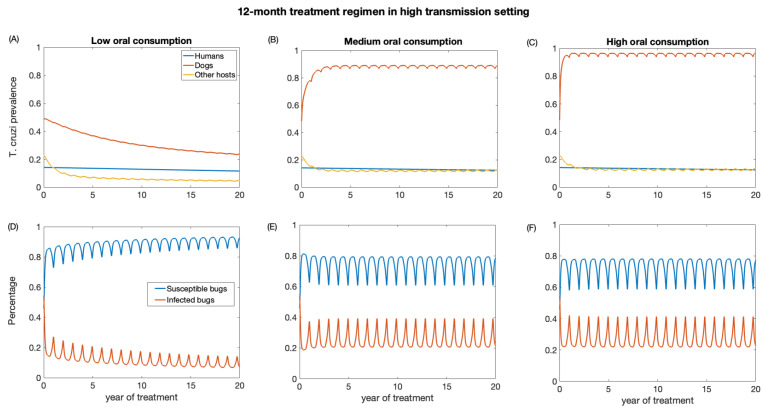
Impact of systemic insecticide treatment of dogs with fluralaner for the control of Chagas disease in high transmission setting with a 12-month regimen under different levels of dog consumption of dead triatomines. (**A**,**D**) Low oral consumption, (**B**,**E**) medium oral consumption, and (**C**,**F**) high oral consumption.

We also investigate the impact of annual treatment (12-month regimen) in the two transmission settings over a period of 20 years. Similar to the 3-month regimen, there was a prompt decline in *T. cruzi* prevalence in both dogs and other host population following the initiation of annual treatment when dogs’ consumption of killed triatomine is very low (Figure 5 and Figure 6). The observed annual transient rebound in *T. cruzi* prevalence was due to the fact that the efficacy of fluralaner for killing triatomines progressively declines at seven months following dog treatment [34,59].

The reduction in *T. cruzi* prevalence in humans under annual treatment was similar to that of the 3-month regimen. In the low-transmission setting, the prevalence of *T. cruzi* in humans was reduced from 0.05 to 0.04, 0.046, 0.047 for low, medium, and high oral consumption of triatomines, respectively (Figure 5), and in a high-transmission setting, the prevalence of *T. cruzi* in humans was reduced from 0.14 to 0.11, 0.12, 0.13 for low, medium, and high oral consumption of triatomines, respectively (Figure 6). For other host populations, the prevalence of *T. cruzi* in the low-transmission setting decreased from 0.08 to 0.008 for low, medium, and high oral consumption with the highest reduction observed immediately after treatment initiation. *T. cruzi* prevalence among dogs decreases from 0.23 to 0.06 in the low transmission setting, while in the high transmission setting, it decreases from 0.48 to 0.24.

With medium and higher consumption of dead triatomines by dogs, the *T. cruzi* prevalence among dogs increases in both transmission settings (Figure 5 and Figure 6). With a medium oral consumption of dead triatomines by dogs (20%), *T. cruzi* prevalence among dogs rises from 0.23 to 0.81 in a low-transmission setting and from 0.48 to 0.89 in a high transmission setting. For a higher oral consumption of dead triatomines (60%), *T. cruzi* prevalence among dogs increases from 0.23 to 0.93 in the low-transmission setting and from 0.48 to 0.96 in the high transmission setting.

Canine fluralaner treatment administered annually was shown to substantially reduce *T. cruzi* prevalence among triatomines in low and high transmission settings when the oral consumption is low. In the low transmission setting, for low oral consumption of triatomines, the percentage of infected triatomine population decreases from 25% to 2%. Similarly, in the high transmission setting, with a low level of oral consumption of triatomines, the percentage of the triatomine population declines from 53% to 6%. With a medium oral consumption of dead triatomines by dogs, the percentage of infected triatomines in the low transmission setting initially decreases from 25% to 17% and subsequently increases to 33% after 3 years of treatment, whereas in a high transmission setting it reduces from 53% to 20% immediately after the first round of treatment and then rises to 39%. With higher oral consumption of dead triatomines, the percentage of infected triatomines decreases in the low transmission setting from 25% to 19% immediately after treatment initiation and then rises to 33%, whereas in the high transmission setting the percentage of infected triatomines initially decreases from 53% to 22% and subsequently increases to 41%.

**Figure 7:**
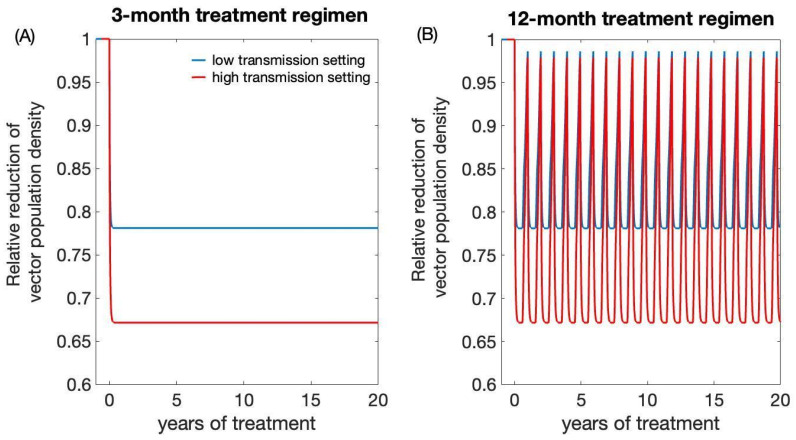
Impact of systemic insecticide treatment of dogs with fluralaner for reducing triatomines population density. (**A**) 3-month treatment regimen, (**B**) 12-month treatment regimen.

In all transmission settings, we observed a prompt decline in vector population density following the initiation of treatment for both regimens (Figure 7). Under the 3-month treatment regimen, the triatomines population was reduced and maintained to 67% of its pre-intervention level in high transmission settings and 79% in low transmission settings (Figure 7A). Under a 12-month regimen, the triatomine population was shown to be initially reduced to 67% of its pre-intervention level in high transmission setting and 79% in low transmission setting after each round of treatment (Figure 7B). However, the triatomine population was shown to recover promptly as fluralaner efficacy wanes (Figure 7B).

Finally, we evaluated the impact of the dogs’ treatment regimens on *T. cruzi* infection incidence among humans (Table 2). We showed that over the first 10 years of treatment, annual dogs’ treatment may reduce *T. cruzi* infection incidence among humans by 74% and 77% in high and low transmission settings, respectively, whereas a three-month treatment regimen may reduce incidence by more than 80% in both transmission settings. However, the effectiveness of the dog treatment regimen for reducing disease incidence among humans varies significantly with the level of additional dog consumption of dead bugs following treatment initiation (Table 2). For example, if dogs eat 20% of dead triatomines (medium consumption), annual dog treatment will reduce *T. cruzi* infection incidence by 52% over the first 10 years in high transmission settings, and by 25% in low transmission settings. A three-month treatment regimen will reduce human infection incidence by 62% in high transmission settings, and by 38% in low transmission settings.

## 4. Discussion

The study evaluated the impact of fluralaner treatment for the control of Chagas disease in different *T. cruzi* transmission settings in the tropics. The effectiveness of treatment was shown to vary significantly with the level of oral consumption of dead triatomine by dogs after treatment initiation. At low oral consumption levels (e.g., 1%), our model showed that dog treatment (once every 3 or 12 months) with fluralaner could substantially reduce *T. cruzi* infection in most hosts including humans and other hosts (rodents). At moderate or high oral consumption of dead triatomines by dogs (e.g., 5% or more), dog treatment may exacerbate *T. cruzi* infection in dogs, and moderately reduce infection in triatomines and other hosts (rodents). In humans, Chagas disease incidence could be reduced by 50–60% in high transmission settings, and by less than 40% in low transmission settings, with effectiveness decreasing with increasing oral consumption rates.

The results of our study have important implications for public health strategies aimed at controlling *T. cruzi* transmission. First, canine fluralaner treatment can effectively reduce *T. cruzi* infection in dogs if canine treatment does not result in a substantial increase in dog consumption of dead triatomines. The effect of systemic insecticide treatment on canine consumption of triatomine remains unknown. To more accurately estimate the effectiveness of systemic insecticide use on *T. cruzi* infection in dogs and humans, empirical field studies are needed to better understand canine triatomine feeding behavior and the impact of systemic insecticide use on this feeding behavior. Although canine treatment can be an effective tool for Chagas disease control, it is important to recognize that reliance on canine treatment alone may not be sufficient to achieve elimination of *T. cruzi* transmission. Additional measures such as insecticide spraying, vaccines, health education, and screening of at-risk human populations are essential components of an integrated approach to Chagas disease control.

As with all mathematical models, our model has some limitations. These limitations are likely to affect our estimates of the impact of dog treatment on reducing *T. cruzi* infection among humans and other hosts. This is due to several simplifying assumptions made. First, our model does not explicitly account for triatomine migration, which may help to replenish the triatomine population in the area of interest and provide an external source of infection. Second, our model does not explicitly account for the fact that some triatomines may not feed on dogs or may not feed continuously on all hosts. The *T. infestans* are opportunistic feeders with nocturnal feeding behavior, so their feeding patterns may be highly heterogeneous among hosts and over time. This may reduce the effectiveness of dog treatment for preventing *T. cruzi* infection among other hosts and humans. Third, our model does not account for triatomine reproductive senescence and seasonality of their feeding patterns, which may also influence the effectiveness of dog treatment for reducing *T. cruzi* transmission dynamics [60]. Fourth, our model does not account for the impact of possible natural or treatment-induced recovery among infected hosts. The presence of recovered hosts will hinder disease transmission and subsequently reduce the effectiveness of systemic insecticide treatment of dogs for control of Chagas disease. However, low recovery rates are anticipated to have minimal impact on the effectiveness of treatment regimens. In addition to ignoring the potential impact of host recovery, our model did not investigate the impact of dogs’ lifespan on disease transmission. Future studies should investigate the impact of these factors on the effectiveness of systemic insecticide treatment of dogs for control of Chagas disease.

Our model made additional assumptions. We grouped hosts into a single infected class, combining the acute and chronic phases of infection, which have different infectivity. We also ignore disease-induced mortality by assuming that hosts could die via natural death. Although such assumptions are expected to have minimal impact on our results, they remain a simplifying assumption. Finally, we assumed that oral transmission occurred only after the bugs were killed by treatment (fluralaner administration). Thus, we considered that there was no significant oral transmission of *T. cruzi* in the dog population prior to the administration of the fluralaner.

## 5. Conclusions

Our study provides valuable insights into the impact of canine fluralaner treatment on *T. cruzi* infection incidence in different host populations and transmission settings. While canine treatment was shown to be effective in directly reducing infections in the dog population, a potential indirect consequence of treatment via increased oral consumption of dead bugs by dogs may result in increased *T. cruzi* infection in dogs. The direct impact of dog treatment on human and other hosts (rodents) infection rates has been shown to vary significantly with the underlying transmission setting and the oral infection rate of dogs. Integrated control strategies that include multiple interventions, including vector control and community engagement, are essential to achieve meaningful reductions in *T. cruzi* transmission and improve public health outcomes. Further research and collaboration between researchers, health authorities, and local communities are essential to develop and implement comprehensive approaches to effectively control Chagas disease in the tropics.

## Figures and Tables

**Table 1:** Parameter table.

Parameter	Description	Values	Reference
$\beta_{OV}$	The transmission rate of *T. cruzi* from triatomine to other hosts	H: 0.000087/day L: 0.000058/day	Estimated from [46,47]
$\beta_{HV}$	The transmission rate of *T. cruzi* from triatomine to humans	H: 0.0000012/day L: 8 × 10^−8^/day	Estimated from [47,48,49]
$\beta_{DV}$	The transmission rate of *T. cruzi* from triatomine to dogs	H: 0.000025/day L: 0.000017/day	Estimated from [47,49,50]
$\beta_{VD}$	The transmission rate of *T. cruzi* from dogs to triatomine	H: 0.0086/day L: 0.0057/day	Estimated from [27,47,49]
$\beta_{VH}$	The transmission rate of *T. cruzi* from humans to triatomine	H: 0.000173/day L: 0.00011/day	Estimated from [47,49,50]
$\beta_{VO}$	The transmission rate of *T. cruzi* from other hosts to triatomine	H: 0.00754/day L: 0.005/day	[47,49,50]
$\mu_{H}$	Human death rate	0.00003641/day	[16]
$\mu_{D}$	Dog death rate	0.000455675/day	[51]
$\mu_{O}$	Other competent death rate	0.0027/day	[2]
$\mu_{V}$	Triatomine death rate	0.005/day	[52]
κ	Carrying capacity of vectors per host	Estimated	From this study
R	The birth rate at carrying capacity	0.09	[53]
ε	Transmission efficiency from infectious triatomine to susceptible dog via oral transmission	0.1	[2]
$N_{V}$	Triatomine population density	31,600 vec/km^2^	[2]
$b_{VD}$	Probability of triatomine infection per feed on dogs	0.3082	[27]
α	Triatomine bites rate	0.1428/day	[47]

**Table 2:** Percentage reduction in *T. cruzi* infection incidence among humans during the first 10 years of treatment. In the absence of dog treatment, the baseline annual endemic incidence was 6.7 cases/10,000 persons per year in low transmission setting and 18.9 cases/10,000 persons per year in high transmission setting.

Transmission Settings	Treatment Frequency					
	Every Three Months			Every Twelve Months		
	Dead Triatomine Consumption			Dead Triatomine Consumption		
	Low	Medium	High	Low	Medium	High
High	80.40%	61.70%	58.90%	74.00%	52.60%	49.30%
Low	81.90%	37.80%	26.30%	76.80%	25.50%	11.20%

## Data Availability

Not applicable.
